# Chemical Characterization of a Renoprotective Metabolite from Termite-Associated *Streptomyces* sp. RB1 against Cisplatin-Induced Cytotoxicity

**DOI:** 10.3390/ijms19010174

**Published:** 2018-01-07

**Authors:** Dahae Lee, Ki Sung Kang, Hae-Jeung Lee, Ki Hyun Kim

**Affiliations:** 1 School of Pharmacy, Sungkyunkwan University, Suwon 16419, Korea; pjsldh@naver.com; 2 College of Korean Medicine, Gachon University, Seongnam 13120, Korea; kkang@gachon.ac.kr; 3 Department of Food and Nutrition, Gachon University, Seongnam 13120, Korea

**Keywords:** *Streptomyces* sp. RB1, 1-*O*-(2-aminobenzoyl)-α-l-rhamnopyranoside, LLC-PK1 cells, renoprotective effect, nephrotoxicity, MAPKs

## Abstract

Platinum-based anticancer drug therapies can cause renal damage and apoptotic kidney cell damage. The development of reno- and kidney-protective molecules is therefore urgently required. To address this challenge, we explored secondary metabolites of termite-associated *Streptomyces* sp. RB1 isolated from the cuticle of the South African termite, *Macrotermes natalensis* for their renoprotective ability using bioassay-guided fractionation and LLC-PK1 cells. Chemical investigation of the MeOH extract of *Streptomyces* sp. RB1 resulted in the isolation and identification of a renoprotective metabolite, 1-*O*-(2-aminobenzoyl)-α-l-rhamnopyranoside (ABR) (**1**) from the active fraction, which ameliorated cisplatin-induced cytotoxicity to 80% of the control value at 25 μM. Upregulated phosphorylation of c-Jun N-terminal kinases (JNK) and p38 following cisplatin treatment was markedly decreased after pre-treatment of cells with ABR. In addition, levels of cleaved caspase-3 and the percentage of apoptotic cells were also significantly reduced after pre-treatment with ABR. These findings provide experimental evidence that blocking the MAPK signaling cascade plays a critical role in mediating the renoprotective effect of ABR, which may inspire the development of novel therapeutic substances to prevent anticancer drug-induced nephrotoxicity.

## 1. Introduction

The platinum chemotherapeutic agent cisplatin is an effective and widely used anticancer drug for treatment of many solid-organ cancers [1]. Unfortunately, cisplatin has serious side-effects, including ototoxicity, gastrotoxicity, myelotoxicity, and allergic reactions. Of these, the significant limiting adverse effect of cisplatin is nephrotoxicity, which is observed in 30% of all cisplatin-treated patients. This nephrotoxicity is dose-dependent, and therefore patients cannot continue being treated with cisplatin once this condition develops [2,3,4,5,6,7,8].

Cisplatin has been on the market since 1978, and kidney-protective therapeutic compounds, enzymes and molecular alterations that help decrease the nephrotoxicity of cisplatin have been identified [7,9]. The underlying mechanisms of these compounds, however, remain unclear. Experimental exposure of kidney cells to cisplatin at concentrations ranging from 16 to 300 μM activates signaling pathways including cell death-promoting mitogen-activated protein kinase (MAPKs), p53, and caspases [10,11].

The research focus in our lab has been secondary metabolites of insect-associated bacteria; in particular, Actinobacteria associated with the fungus-growing termite *Macrotermes natalensis* [12,13,14]. Termite-associated bacteria have previously been shown to produce structurally interesting and bioactive secondary metabolites such as microtermolides A and B [13]. Our initial study of termite-associated bacteria led to the discovery of a highly unusual geldanamycin analogue, natalamycin A, from termite-associated *Streptomyces* sp. M56 [12]. Recently, we explored termite-associated *Amycolatopsis* sp. M39, which resulted in the characterization of four new 20-membered glycosylated polyketide macrolactams, macrotermycins A–D [14]. In an effort to extend earlier studies, we investigated the termite-associated isolate *Streptomyces* sp. RB1, which was isolated from the cuticle of a *Macrotermes natalensis* worker collected in South Africa in 2015. We identified three new isoflavonoid glycosides, termisoflavones A–C, and investigated their antifungal and antibacterial activities [15]. As part of our continuing efforts to study the bioactive secondary metabolites of termite-associated *Streptomyces* sp. RB1, we found that the MeOH extract of *Streptomyces* sp. RB1 showed a protective effect against cisplatin-induced cytotoxicity, which led us to investigate the renoprotective metabolites in the MeOH extract using a bioassay-guided fractionation method in LLC-PK1 cells. Here, we describe the isolation and structural elucidation of a renoprotective metabolite (**1**), as well as its renoprotective effects against cisplatin-induced cytotoxicity and its protective mechanism of action.

## 2. Results

### 2.1. Bioactivity-Guided Fractionation and Isolation of a Renoprotective Metabolite

**Figure 1:**
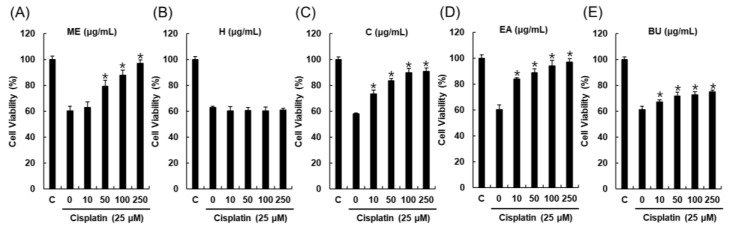
Protective effects of the MeOH extract (ME) of *Streptomyces* sp. RB1 and its fractions (hexane-soluble (H), CH_2_Cl_2_-soluble (C), EtOAc-soluble (EA), and *n*-BuOH-soluble fractions (BU)) in LLC-PK1 cells exposed to 25 μM of cisplatin for 24 h by MTT assay. Protective effects of (**A**) ME, and (**B**) H fraction, (**C**) C fraction, (**D**) EA fraction, and (**E**) BU fraction in LLC-PK1 cells exposed to 25 μM of cisplatin for 24 h by MTT assay. The concentrations of samples were 0, 10, 50, 100 and 250 μg/mL. Control cells were treated with the vehicle only (mean ± SD, * *p* < 0.05 compared to the control).

**Figure 2:**
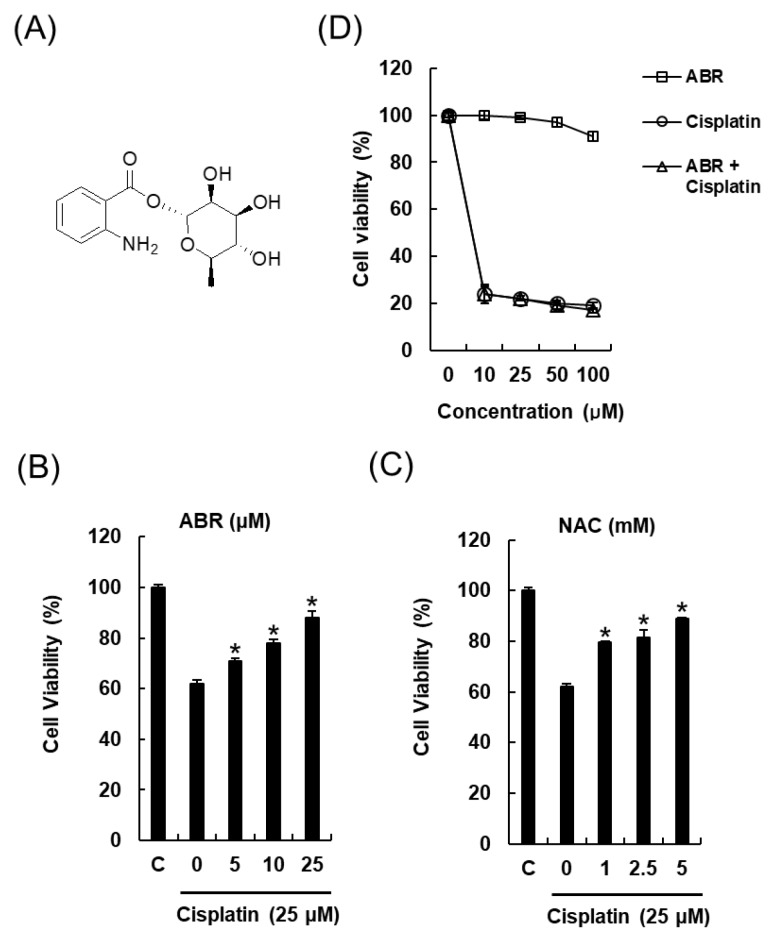
Protective effect of 1-*O*-(2-aminobenzoyl)-α-l-rhamnopyranoside (ABR) in LLC-PK1 cells exposed to 25 μM of cisplatin for 24 h by MTT assay. (**A**) Chemical structure of ABR. Dose-dependent protective effects of (**B**) ABR and (**C**) *N*-acetyl cysteine (NAC) in LLC-PK1 cells exposed to 25 μM of cisplatin for 24 h by MTT assay; (**D**) Combined effects of ABR and cisplatin on the cell viability in human ovarian cancer A2780 cells. NAC was used as a positive control. Control cells were treated with the vehicle only (mean ± SD, * *p* < 0.05 compared to the control).

*Streptomyces* sp. RB1 was grown on 60 ISP-2 agar plates and the agar was extracted with MeOH to obtain a crude MeOH extract (ME). The ME showed a renoprotective effect against cisplatin-induced cytotoxicity to 79.2% ± 4.7% of the control value at 50 μg/mL (Figure 1A). ME was successively solvent-partitioned with hexane, CH_2_Cl_2_, EtOAc, and *n*-BuOH to give four main fractions [hexane-soluble (H), CH_2_Cl_2_-soluble (C), EtOAc-soluble (EA), and *n*-BuOH-soluble fractions (BU)]. The kidney protection effects of the four main fractions (H, C, EA, and BU) were evaluated in LLC-PK1 cells to identify active fractions. Of the fractions tested, the EA fraction was the most active fraction and ameliorated cisplatin-induced cytotoxicity to 83.9% ± 0.9% of the control value at 10 μg/mL (Figure 1D). The C fraction showed a moderate protective effect, but this was not statistically significant (Figure 1C). Based on this result, we investigated the EA fraction for renoprotective metabolites. Chemical investigation of the EA fraction using column chromatography and HPLC purification led to the isolation and identification of a renoprotective metabolite (**1**), which was identified as 1-*O*-(2-aminobenzoyl)-α-l-rhamnopyranoside (ABR) by comparing the spectroscopic data with previously reported values and LC/MS analysis (Figure 2) [16,17].

### 2.2. Protective Effects of 1-O-(2-aminobenzoyl)-α-l-rhamnopyranoside (ABR) in LLC-PK1 Cells Exposed to 25 μM of Cisplatin for 24 h by MTT Assay

LLC-PK1 cells damaged by cisplatin were used to examine the renoprotective effect of 1-*O*-(2-aminobenzoyl)-α-l-rhamnopyranoside (ABR). The results are presented in Figure 2. Treatment of cells for 24 h with 25 μM cisplatin caused a 62.02% ± 1.34% reduction in cell viability compared to untreated controls. Pre-treatment for 2 h with ABR at different concentrations had a significant protective effect against cell damage caused by cisplatin, and ABR (25 μM, 88.15% ± 2.60%) had a much greater effect than the positive control, *N*-acetyl cysteine (NAC) (5 mM, 88.96% ± 0.25%). To evaluate the effect of ABR on the antitumor activity of cisplatin, human ovarian cancer A2780 cells were treated with ABR and cisplatin alone or in combination. Co-treatment with ABR at a dose of up to 100 μM slightly increased cytotoxic property of cisplatin (Figure 2D), but ABR alone did not attenuate it. Our findings suggest that ABR with cisplatin may alleviate the nephrotoxicity without compromising therapeutic efficiency of cisplatin.

### 2.3. Effects of ABR on Apoptosis in LLC-PK1 Cells Exposed to Cisplatin

**Figure 3:**
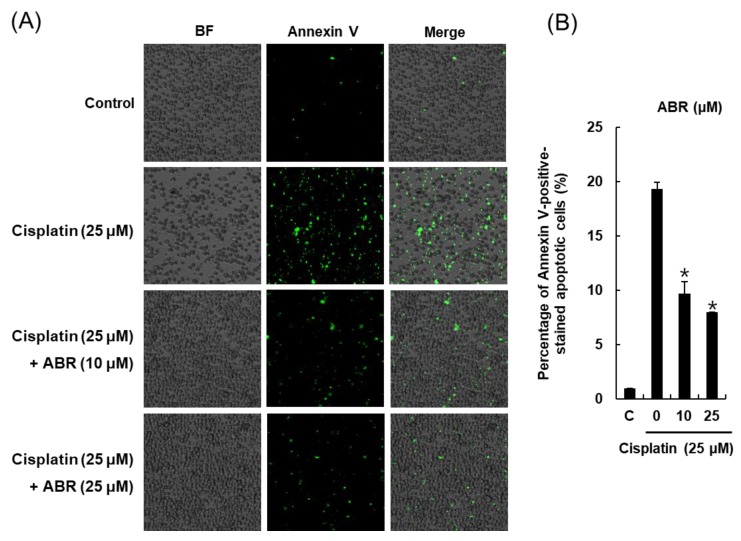
Protective effect of ABR on apoptosis in LLC-PK1 cells exposed to 25 μM cisplatin for 24 h by image-based cytometric assay. (**A**) Representative images for apoptosis detection (green) (Magnification: 4×); (**B**) Percentage of Annexin V-positive-stained apoptotic cells. Control cells were treated with the vehicle only (mean ± SD, * *p* < 0.05 compared to the control).

We further explored whether ABR could decrease apoptosis in LLC-PK1 cells exposed to cisplatin through annexin V Alexa Fluor 488 staining. As shown in Figure 3, apoptotic cells were stained green with annexin V Alexa Fluor 488. Bright-field (BF) and merge images are also shown in Figure 3A. The percentage of Annexin V-positive-stained apoptotic cells is shown in the bar graph. The percentage of apoptotic cells was increased by 25 μM cisplatin from 1.00% ± 0.00% to 19.33% ± 0.57%, whereas it was decreased by 9.66% ± 1.15% and 8.00% ± 0.00% when cells were pretreated with 10 or 25 μM ABR, respectively (Figure 3B).

**Figure 4:**
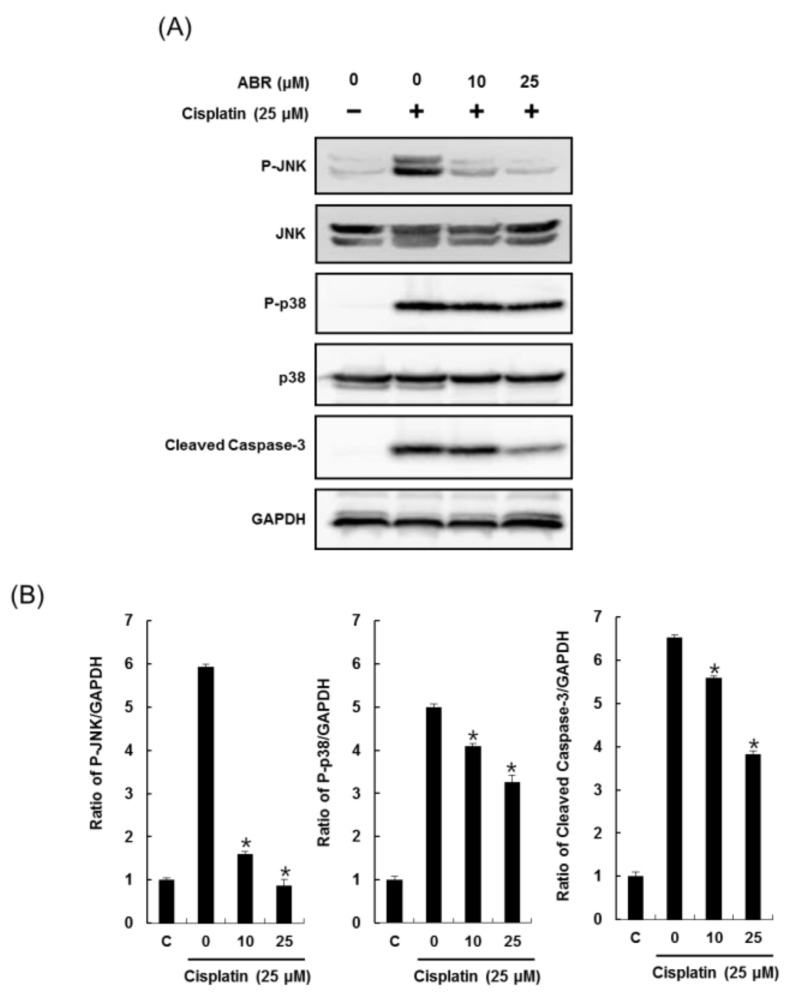
Protective effect of ABR on apoptosis in LLC-PK1 cells exposed to 25 μM cisplatin for 24 h by western blot (**A**) Expression levels of MAPK-caspase-3 pathway proteins; (**B**) Each bar graph represents densitometric quantification of western blot bands. Control cells were treated with the vehicle only (mean ± SD, * *p* < 0.05 compared to the control).

The effects of 10 and 25 μM ABR on expression of phospho-JNK, JNK, phospho-p38, p38 and cleaved caspase-3 in LLC-PK1 cells exposed to cisplatin were evaluated through western blots (Figure 4).

Levels of phosphorylated JNK, phosphorylated p38, and cleaved caspase-3 were increased by 25 μM cisplatin treatment, whereas they were decreased by pretreatment of cells with 10 and 25 μM ABR in a dose dependent manner (Figure 4A). Bar graphs show levels of phosphorylated JNK, phosphorylated p38, and cleaved caspase-3 expression normalized by GAPDH (Figure 4B).

## 3. Discussion

Our major findings are that ABR treatment attenuated the direct toxic effect of cisplatin on proximal tubular cells and the apoptotic pathway, including the MAPK signaling pathway. In the kidney, cisplatin, which is an uncharged, low-molecular weight molecule, is freely filtered at the glomerulus and it is taken up specifically by renal tubular cells. Therefore, the cellular pathways of cisplatin-induced renal injury to kidney cells have been examined primarily in renal tubular cells [5,18,19].

Treatment of LLC-PK1 cells with cisplatin for 24 h markedly reduced cell viability. Pre-treatment with ABR protected against the cisplatin-induced cell damage. This result indicates that ABR is capable of protecting LLC-PK1 cells from cisplatin-induced damage. However, cisplatin-induced renal injury through a direct toxic effect on proximal tubular cells is a common histopathological characteristic [4,20]. The mechanisms underlying cisplatin-induced cytotoxicity are very complex and involve a number of interconnected cellular processes including apoptosis, inflammation, oxidative stress, nuclear and mitochondrial DNA damage, and mitochondrial dysfunction and autophagy [5,9,18].

Apoptosis is a regulated and controlled type of cell death that is characterized by DNA fragmentation, cell shrinkage, chromatin condensation, and membrane blistering, and is a final common pathway in response to a variety of cellular stresses. Several apoptosis pathways have been implicated in cisplatin-induced renal injury [9,18]. Apoptotic cell death in LLC-PK1 cells was detected after 12 h of exposure to low concentrations (10–100 μM) of cisplatin, whereas at higher concentrations (200–800 μM), cisplatin mainly induced necrotic cell death in LLC-PK1 cells [3,9]. In the present study, a much lower concentration of cisplatin (25 μM) was used, which also induced significant apoptosis in LLC-PK1 cells. This might be due to the longer treatment time (24 h) used in the present study compared to the previous study. Pre-treatment with ABR effectively attenuated cisplatin-induced cell apoptosis. This result was in accordance with the improvement in cell viability of ABR-treated cells, suggesting that the protective effect of ABR on LLC-PK1 cells might be derived from inhibition of cell apoptosis by cisplatin. Cisplatin-induced apoptosis was further confirmed by activation of cleaved caspase-3 activation and phosphorylation of JNK and p38. A variety of biological, physical, and chemical cellular stresses activate mitogen-activated protein kinase (MAPK) intracellular signaling proteins such as extracellular signal-regulated kinases (ERK), p38, and c-Jun N-terminal kinase (JNK) [19]. Specific inhibition of MAPKs reduces caspase activation, apoptosis, the inflammatory response, and injury to the kidney both in vitro and in vivo [4,5,18,21]. Inhibitors of p38 or JNK can attenuate acute renal failure, tubular cell apoptosis, renal ischemia/reperfusion, and histologic damage induced by cisplatin [21,22,23]. In the present study, exposure to cisplatin induced significant phosphorylation of JNK and p38 in LLC-PK1 cells, while pre-treatment with ABR effectively reduced phosphorylation of JNK and p38.

Activation of a family of apoptotic proteases that includes caspase-3, -6, -7, -8, -9, and -10 is important in the genesis of apoptosis in cisplatin-induced renal tubular epithelial cell death [3,5]. Initiator caspases (caspase-8, and -9) involved in cleaving effector caspases (caspase-3, and -7) are activated by various apoptotic stimuli including reactive oxygen species, ischemia, and stress [24,25]. Among these, caspase-3 is a major apoptotic protease that acts in the final pathway of apoptosis and is involved in cleaving important substrates, such as poly(ADP-ribose) polymerase (PARP), in nuclear processes [7,26]. In the present study, exposure to cisplatin resulted in increased levels of cleaved caspase-3 in LLC-PK1 cells, while pre-incubation with ABR effectively reduced the levels of cleaved caspase-3. Pre-treatment with ABR protected against cell damage and significantly reversed cisplatin-induced phosphorylation of JNK and p38 and enhancement in levels of cleaved caspase-3, suggesting that ABR inhibited cisplatin-induced LLC PK1 cell apoptosis. These results are consistent with previous studies that reported that cisplatin-induced LLC PK1 cell damage was suppressed by inhibiting cleavage of caspase-3 [27,28,29] and phosphorylation of JNK and p38 [28,30,31]. These findings suggesting that the suppressive effect of ABR on cleaved caspase-3 levels and the phosphorylation of JNK and p38 might be mediated by its antioxidant capacity, allowing it to effectively scavenge cisplatin-induced reactive oxygen species.

## 4. Materials and Methods

### 4.1. General Experimental Procedures

Specific rotations were measured on a Jasco P-1020 polarimeter (Jasco, Tokyo, Japan). IR spectra were recorded on a Bruker IFS-66/S FT-IR spectrometer (Bruker, Karlsruhe, Germany). UV spectra were acquired on an Agilent 8453 UV-visible spectrophotometer (Agilent Technologies, Milford, MA, USA). Liquid chromatography/mass spectrometry (LC/MS) analysis was performed on an Agilent 1200 Series HPLC system (Agilent Technologies) equipped with a diode array detector and a 6130 Series ESI mass spectrometer using an analytical Kinetex C18 100 Å column (100 mm × 2.1 mm i.d., 5 μm). NMR spectra were recorded on a Bruker AVANCE III 700 NMR spectrometer operating at 700 MHz (^1^H) and 175 MHz (^13^C). A Shimadzu Prominence HPLC System with SPD-20A/20AV Series Prominence HPLC UV-Vis detectors (Shimadzu, Tokyo, Japan) was used for semi-preparative HPLC. Column chromatography was performed with silica gel 60 (Merck, 230–400 mesh, Frankfurt, Germany) and RP-C18 silica gel (Merck, 230–400 mesh). The packing material for molecular sieve column chromatography was Sephadex LH-20. Merck pre-coated silica gel F254 plates and reversed-phase (RP)-18 F254s plates were used for thin layer chromatography (TLC). Spots on TLC plates were detected under UV light or by heating the plates after spraying with anisaldehyde-sulfuric acid.

### 4.2. Extraction and Isolation

*Streptomyces* sp. RB1 was isolated from the cuticle of a *M. natalensis* worker collected in South Africa, and its sequencing and species identification were verified [15]. *Streptomyces* sp. RB1 was grown on 60 ISP-2 agar plates (9 cm diameter) for 14 days at 30 °C. Agar was cut into squares, consolidated, and soaked overnight in MeOH. The MeOH phase was filtered and the solvent removed under reduced pressure to give a crude MeOH extract (20 g). The MeOH extract (ME) showed a renoprotective effect against cisplatin-induced cytotoxicity to approximately 80% of the control value at 50 μg/mL (Figure 1A). ME was suspended in distilled water (250 mL) and then successively partitioned with hexane (H), CH_2_Cl_2_ (C), ethyl acetate (EA), and *n*-BuOH (BU), yielding 0.06, 0.32, 0.35, and 1.57 g of residue, respectively. Kidney protective effects of H, C, EA, and BU fractions were evaluated in LLC-PK1 cells. Of the fractions tested, the EA fraction was the most active fraction and ameliorated cisplatin-induced cytotoxicity to 83.9% ± 0.9% of the control value at 10 μg/mL (Figure 1D). Therefore, the EA fraction was investigated to identify compounds with a potential renoprotective effect. The EA fraction (0.35 g) was applied to a Sephadex LH-20 column (50%–100% MeOH) for chromatographic separation to give 14 subfractions (E1–E14). Subfraction E10 (13 mg) was purified by semi-preparative reversed-phase HPLC using a gradient program (water [A], methanol [B]: 0–23 min: 42% B; 23–40 min: 47% B) with a phenyl-hexyl column (Phenomenex, Luna, 250 × 10 mm, i.d., 5 μm) and a flow rate of 2 mL/min to yield compound **1** (1.3 mg, *t*_R_ = 20.5 min).

### 4.3. Cell Culture and MTT Cell Viability Assay

The protective effect of the test samples against cisplatin-induced renal cell damage was identified in LLC-PK1 pig kidney epithelium cells purchased from the American Type Culture Collection (ATCC, Manassas, VA, USA). These cells were cultured in Dulbecco’s modified Eagle medium (ATCC) supplemented with 10% fetal bovine serum (Invitrogen, Grand Island, NY, USA), 1% penicillin/streptomycin, and 4 mM l-glutamine in a humidified incubator with 5% CO_2_ in air at 37 °C. Cells were split on a weekly basis at a ratio of 1:5 upon reaching confluency. Cells were seeded at a density of 1 × 10^4^ cells per/well in 96-well culture plates. One day after seeding, cells were treated for 2 h with the test samples. Then, 25 μM cisplatin was added to wells for 24 h. Control cells were treated with the vehicle only. After incubation, the Ez-Cytox assay was carried out to determine cell viability. Briefly, medium containing the test samples and/or 25 μM cisplatin was removed, and cells were incubated in serum-free medium (90 μL/well) with Ez-Cytox reagent (10 μL/well) for 2 h at 37 °C. Cell viability was measured by absorbance at 450 nm using a microplate reader (PowerWave XS; Bio-Tek Instruments, Winooski, VT, USA) [32,33,34,35,36]. *N*-acetyl cysteine (NAC) was used as a positive control.

To evaluate the effect of compound on the antitumor activity of cisplatin, human ovarian cancer A2780 cells were treated with the compound and cisplatin alone or in combination. The A2780 human ovarian carcinoma cell line was purchased from the American Type Culture Collection (ATCC) and maintained in Roswell Park Memorial Institute 1640 medium (RPMI 1640) (Cellgro, Manassas, VA, USA) supplemented with 10% fetal bovine serum (Gibco BRL, Gaithersburg, MD, USA), 100 units/mL penicillin, and 100 mg/mL streptomycin with incubation at 37 °C in a humidified atmosphere with 5% CO_2_. The A2780 cells were seeded at 1 × 10^4^ cells/100 μL in 96-well plates. After incubation for 24 h, the cells were incubated in cell culture medium with or without test samples for a further 24 h. Cell viability was determined using the Ez-Cytox cell viability assay kit (Daeil Labservice, Seoul, Korea) according to the instructions.

### 4.4. Image-Based Cytometric Assay

Cells were seeded at a density of 4 × 10^5^ cells per/well in 6-well plates. One day after seeding, cells were treated for 2 h with the samples. Then, 25 μM cisplatin was added to wells for 24 h. Control cells were treated with the vehicle only. After incubation, cells were washed twice with cold phosphate-buffered saline and scraped off the plates. Cell apoptosis was determined after annexin V Alexa Fluor 488 staining (Invitrogen, Temecula, CA, USA) using a Tali image-based cytometer (Invitrogen, Temecula, CA, USA).

### 4.5. Western Blotting Analysis

Cells were seeded at a density of 4 × 10^5^ cells per/well in 6-well plates. One day after seeding, cells were treated for 2 h with the test samples. Then, 25 μM cisplatin was added to wells for 24 h. Untreated cells were used as controls. After incubation, cells were washed twice with cold phosphate-buffered saline and scraped off the plates, and then lysed using RIPA buffer (Cell Signaling Technology, Inc., Beverly, MA, USA) supplemented with 1× EDTA free protease inhibitor cocktail and 1 mM phenylmethylsulfonyl fluoride (PMSF) according to the manufacturer’s instructions. Aliquots of 20 μg protein were subjected to SDS PAGE (10% gels) along with molecular weight markers for 90 min at 110 V, and then transferred to PVDF transfer membranes. Membranes were probed with primary antibodies to phospho-JNK, JNK, phospho-p38, p38, cleaved caspase-3, GAPDH and then with secondary immunoglobulin G horseradish peroxidase conjugates. Bound antibodies were detected using ECL Advance Western Blotting Detection reagents (GE Healthcare, Cambridge, UK) and a FUSION Solo Chemiluminescence System (PEQLAB Biotechnologie GmbH, Erlangen, Germany).

### 4.6. Statistical Analysis

All data, including cell viability, percentage of apoptotic cells and protein expressions were presented as the average value and standard deviation (SD). All the assays were done in triplicate for each assay and were repeated at least three times. In this study, only a small number of repetitions for each cell experiment was included; thus, a non-parametric analysis method was adopted for statistical analysis. The Kruskall-Wallis test was used for the statistical analysis of each variable. SPSS statistical package was used for all analyses (IBM SPSS statistics version 21, Boston, MA, USA). Statistical significance was considered at a *p*-value lower than 0.05.

## 5. Conclusions

In the present study, we found that the MeOH extract of *Streptomyces* sp. RB1 had a protective effect against cisplatin-induced cytotoxicity, which led us to attempt to isolate renoprotective metabolites from the MeOH extract using a bioassay-guided fractionation method in LLC-PK1 cells. We identified a renoprotective metabolite, 1-*O*-(2-aminobenzoyl)-α-l-rhamnopyranoside (ABR), from the active EA fraction that at a concentration of 25 μM ameliorated cisplatin-induced cytotoxicity to 80% of the control value. Investigation of the protective mechanism of action demonstrated that ABR was capable of suppressing cisplatin-induced LLC-PK1 cells apoptosis through inhibition of the phosphorylation of JNK and p38 and cleavage of caspase-3. These observations provide insight into the mechanisms involved in cisplatin-induced LLC-PK1 damage. However, more intensive studies are needed to explore additional mechanisms responsible for the renoprotective effects of ABR as an adjunct candidate to treat cisplatin-associated side effects.
